# Bing-Neel Syndrome: Real-Life Experience in Personalized Diagnostic Approach and Treatment

**DOI:** 10.3389/fonc.2022.891052

**Published:** 2022-06-29

**Authors:** Dimitrios Kotsos, Sofia Chatzileontiadou, Athanasia Apsemidou, Anna Xanthopoulou, Aikaterini Rapi, Christina Frouzaki, Evdoxia Hatjiharissi

**Affiliations:** Hematology Unit, 1^st^ Department of Internal Medicine, AHEPA University Hospital, Thessaloniki, Greece

**Keywords:** Waldenström’s Macroglobulinemia, Bing-Neel syndrome, central nervous system infiltration, ibrutinib, real-life data

## Abstract

The involvement of the central nervous system (CNS) in Waldenström’s Macroglobulinemia (WM) is a rare extramedullary manifestation of the disease known as Bing-Neel syndrome (BNS). To expand our understanding of this disease manifestation, we conducted a retrospective analysis of the incidence of BNS in 86 consecutive patients with WM [70% male, median age 65 years (range 33-86)] seen in our center during a 30-year period. Six patients (7%) from this group were diagnosed with BNS. The median period of time between WM diagnosis and BNS diagnosis was 6.8 years (range 2.3-15). They demonstrated a range of neurological deficits, including transient expressive aphasia, impaired vision, resting hand tremor, foot drop, and headache. Between the onset of symptoms and the diagnosis of BNS, the median time interval was 12.5 months (range 1-30). The diagnosis was made not on the basis of neurological symptoms or radiological evidence, but on the basis of the presence of WM cells in cerebrospinal fluid (CSF). Intrathecal chemotherapy with methotrexate, cytarabine, and dexamethasone (IT MTX, ARA-C, DEX) was used as front-line treatment, followed by intensive immunochemotherapy with rituximab, high-dose MTX, and ARA-C (R-Hi MTX/ARA-C) in three patients who were fit enough to receive this type of cytotoxic regimen, and rituximab plus bendamustine (R-Benda) in two patients who simultaneously required treatment for WM. Ibrutinib was administered to five patients (three as consolidation and two for initial treatment). All patients responded to front-line treatment, with four (67%) achieving partial response (PR) and two (33%) achieving complete response (CR). This study provides insight into the clinical presentation, diagnostic and treatment options, as well as the outcome of patients who have BNS.

## Introduction

Bing-Neel syndrome (BNS) is an uncommon extramedullary manifestation of Waldenström’s Macroglobulinemia (WM) resulting from an unexplained migration and homing of clonal lymphoplasmacytic cells (LPCs) to the central nervous system (CNS). It was first described in 1936 by two physicians, Jens Bing and Axel Neel, who reported on two patients with neurological deficits, hyperglobulinemia and the presence of LPCs in the cerebrospinal fluid (CSF) (1). Their description actually predated Jan Waldenström’s characterization of the disease in 1944 (2). Since then, a small number of case reports referring to this unique entity have been published.

BNS is observed in just 1% of patients diagnosed with WM (3, 4), while WM is extremely rare, with an annual incidence of only 2.5 cases per million (5). The clinical picture of BNS is very heterogeneous, lacking specific neurological signs and symptoms. Patients typically present with a variety of neurological deficits, including but not limited to balance disorders, ataxia, sensory and motor deficits, headaches, and cognitive impairment (6, 7). BNS diagnosis entails, in addition to clinical evaluation, the use of imaging studies, CSF analysis, and biopsies. Magnetic resonance imaging (MRI) of the brain and the entire spine should be performed as part of the initial evaluation, as both sites can be affected (8). Patients with BNS often demonstrate two types of CNS involvement: the leptomeningeal type, which is caused by the invasion and circulation of LPCs into the CNS, and a less common type, which is characterized by brain masses (9). Lumbar puncture is a key element for BNS diagnosis, since it identifies CSF leukocytosis and serves as a source for clonal B-cell detection by flow cytometric analysis and/or molecular studies. Recently, the mutation of the *Myeloid differentiation primary response 88 (MYD88 L256P)* gene and the identification of immunoglobulin heavy chain (IgH) locus rearrangements have emerged as promising diagnostic techniques for BNS (10, 11). Hematologists are seldom aware of or prepared for the CNS infiltration of this disease, which is typically characterized by a long, natural course, similar to that of indolent lymphomas (4). For all the above reasons, diagnosis of BNS is rendered a true challenge in everyday medical practice, emphasizing the critical importance of collaborating with Waldenström’s specialists.

CNS invasion is a site of disease that is very difficult to treat across all lymphoma subtypes. Historically, BNS has been treated with chemotherapeutic agents that enter into the CNS. The therapeutic regimens were adapted from the current treatment options for primary CNS lymphomas (PCNSL) with systemic high-dose methotrexate being preferred (12). Recently, the introduction of a new class of drugs, the Bruton tyrosine kinase inhibitors (BTKi), including ibrutinib, has shown promising efficacy in both disease and BNS management, leading to a rapid shift in treatment paradigms for this disease (13).

We provide our real-world experience with BNS, illustrating, through a case series of challenging presentations, the clinical spectrum of the disease. We analyzed the diagnostic challenges of six patients with BNS, who were seen in our center, with the goal of eliciting discussion about the best strategies for managing BNS across the disease continuum.

## Rationale

We conducted a single-center, retrospective study to assess the incidence of BNS in 86 consecutive patients with WM [70% male, median age 65 years (range 33-86)], who were seen in our center between 1991 and 2021. Of these, 6 patients (7%) were diagnosed with BNS. The diagnosis was a multi-step process, including an MRI of the brain and spine in every patient with persistent, unexplained neurological symptoms, followed by a lumbar puncture and CSF examination. CSF analysis included cell count and morphology examination, supplemented by flow cytometric analysis in cases of pleocytosis (more than 5 white blood cells/mm³), along with molecular testing, such as detection of *MYD88* mutation when this became available. Demographic, clinical, radiological and laboratory characteristics were collected and analyzed from the patients’ records. Response to treatment was evaluated based on criteria published by Minnema et al. (9).

## Results

### Patients’ Characteristics

Among 86 consecutive patients with WM, 6 (7%) patients were diagnosed with BNS, all of them men. No woman was presented with this complication, despite the fact that almost one-third of the WM patients were female (26/86). The median age at diagnosis of WM for BNS patients was 57 years (range 33-62). The median age at BNS diagnosis was 62 years (range 48-71). The median time between WM and BNS diagnosis was 6.8 years (range 2.3-15). Interestingly, we observed two different patterns of BNS development defined as follows: early BNS [patients (n=3) who were diagnosed in the next 4.5 years following their WM diagnosis] and late BNS [patients (n=3) diagnosed after at least 9 years of WM diagnosis]. The median time between the onset of symptoms and BNS diagnosis was 12.5 months (range 1-30). Five of them carried the *MYD88 (L256P)* mutation in the bone marrow LPCs, while one patient (case 1) was not tested since at the time of his diagnosis the mutation was not known yet.

Summary of WM and BNS disease characteristics are provided in **Tables 1**, **2**, respectively. Details on WM disease history are provided in **Supplementary Table 1**.

**Table 1:** Summary of WM disease characteristics.

Case number, sex	Age at WM diagnosis	BMI (%)	IgM (g/l)	Treatment for WM prior to BNS	Time (months) from WM diagnosis to BNS onset	Treatment at the time of BNS diagnosis
Case 1, M	62	80	83.0	R-CHOP, Rituximab	31 (PNS), 55 (CNS)	None
Case 2, M	40	60	44.6	DRC, Rituximab, CPA+Dex, DRC, Rituximab	90	Rituximab
Case 3, M	56	65	51.2	Rituximab, Chlorambucil, DRC, FC, R-CEOP	150	None
Case 4, M	61	30	76.7	FCR (R added after IgM reduction)	27	None
Case 5, M	33	90	117.0	Plex plus DRC, CEOP, plex plus FCR	174	None
Case 6, M	58	12	41.6	No prior treatment	27	R-Benda followed by Ibrutinib

M, male; WM, Waldenström’s Macroglobulinemia; BNS, Bing-Neel syndrome; BMI, bone marrow infiltration; IgM, immunoglobulin M; CNS, central nervous system; PNS, peripheral nervous system; R-CHOP, rituximab-cyclophosphamide, doxorubicin hydrochloride, vincristine, prednisone; DRC, dexamethasone, rituximab, cyclophosphamide; CPA, cyclophosphamide; Dex, dexamethasone; FC, fludarabine, cyclophosphamide; FCR, fludarabine, cyclophosphamide, rituximab; R-CEOP, rituximab-cyclophosphamide, etoposide, vincristine, prednisone; R-Benda, rituximab-bendamustine; plex, plasmapheresis.

**Table 2:** Summary of BNS characteristics.

		CSF analysis						
Case Number	Age at BNS diagnosis	BF counts (cells/mm³)	Protein (mg/dl)	Glucose (mg/dl)	FCA	IF	FLCs (fκ/λ)	BNS treatment and response
Case 1	67	33	536	74	monotypic CD19+, CD20+,κ-chain restricted B-cells	IgM-κ	36	IT CHT ×4 R-Hi MTX/ARA-C ×2 (PR) On relapse: IT CHT ×6
Case 2	50	51	118	64	1. monotypic CD19+, CD20+, κ-chain restricted B-cells 2. monotypic CD38+, CD138+, CD56-, κ-chain restricted plasma cells	IgM-κ	117	IT CHT ×4 R-Hi MTX/ARA-C ×3 followed by Ibrutinib 10 months later (PR) On relapse: R-Benda ×6, Venetoclax
Case 3	71	59	433	41	monotypic CD19+,CD20+, CD38+, κ-chain restricted B cells	IgM-κ	261	IT CHT × 4, Ibrutinib (PR) On relapse: IT CHT × 4 Benda × 1
Case 4	63	43	NA	NA	monotypic CD19+, CD20+, CD5+, κ-chain restricted B cells	ND	ND	IT CHT × 4 Ibrutinib (ongoing) plus R-Hi MTX/ARA-C × 4 (PR)
Case 5^*^	48	400	502	NA	monotypic CD19+, CD20+, CD38+, κ-chain restricted B cells	ND	ND	IT CHT × 4 R-Benda × 1 followed by Ibrutinib (ongoing) (CR)
Case 6	61	67	206	61	monotypic CD 19+, CD20+ with selective expression of κ-chain B cells	ND	ND	IT CHT × 4 R-Benda × 6 followed by Ibrutinib (ongoing) (CR)

BNS, Bing-Neel syndrome; CSF, cerebrospinal fluid; BF counts, body fluid counts; FCA, flow cytometric analysis; IF, immunofixation; FLCs, free light chains; fκ/λ, free kappa/lambda; MYD88 mut, Myeloid differentiation primary response 88 mutation; IgM, immunoglobulin M; κ, kappa light chains; NA, not available; ND, not done; IT CHT, intrathecal chemotherapy; MTX, methotrexate; ARA-C, cytarabine; DEX, dexamethasone; R, rituximab; Hi MTX/ARA-C, high dose methotrexate/cytarabine; Benda, bendamustine, CR, complete response; PR, partial response.

^*^MYD88 mutation was detected in the CSF specimen.

### Clinical Picture, Imaging, and Diagnosis

Each patient diagnosed with BNS had a unique clinical presentation. Motor impairments (5/6) and progressive headaches (4/6) were the most frequently reported symptoms, followed by gait disturbance (3/6) and diplopia (3/6). Sensory deficits, including paresthesia (1/6) and hypoesthesia (1/6) were also evident.

**Case 1** presented with left upper limb paresthesia coupled with left palmar interossei muscular atrophy on physical examination. Electromyography (EMG) revealed dysfunction of the left median and ulnar nerves. These findings were interpreted as multiple mononeuropathy syndrome, which may have been caused by WM. However, subsequent testing for anti-MAG antibodies, cryoglobulins, and fat biopsy for amyloid were all negative. The patient developed right foot drop and bilateral lower limb paresthesia two years later. The EMG was repeated and revealed a significant decrease in the conduction velocity of the right peroneal nerve, consistent with localized demyelination and axonal injury. Brain and lumbosacral spine MRI revealed hyperplasia of the lymphoid tissue of the nasopharynx and white lesions at corona radiata bilaterally. In view of the findings, we performed a lumbar puncture, which demonstrated an increased number of monotypic WM cells (33 cells/mm³) in the CSF. Following that, a biopsy of the right peroneal nerve showed no evidence of WM infiltration, focal demyelination, or other aberrant findings. Later in the course of BNS, the patient developed blepharoptosis and diplopia, both of which are signs of oculomotor nerve palsy induced by LPCs invasion.

**Case 2** initially complained of worsening headaches, impaired vision, and right upper limb weakness. He underwent an extensive work-up by neurologists, which included a carotid ultrasound that revealed mild stenosis and a normal magnetic resonance angiography (MRA). His brain MRI revealed a high signal in the subcortical white matter, which was most likely caused by ischemia. He had stable disease at the time, with a serum IgM level of 27 g/l. After 15 months, the patient developed transient expressive aphasia, dysarthria, and gait dysfunction. A repeat brain MRI revealed no change in the findings, and so all symptoms were attributed to a transient ischemic attack (TIA) for which the patient was treated with aspirin. Nonetheless, his symptoms gradually worsened, and he was admitted to the hospital many times for reevaluation. Shortly thereafter, the second diagnosis of seizures was added, along with concurrent antiepileptic therapy. After 19 months of no improvement in symptoms, we became extremely suspicious of CNS infiltration by WM. As a result, we did a lumbar puncture, which verified the presence of clonal LPCs in the CSF.

**Figure 1:**
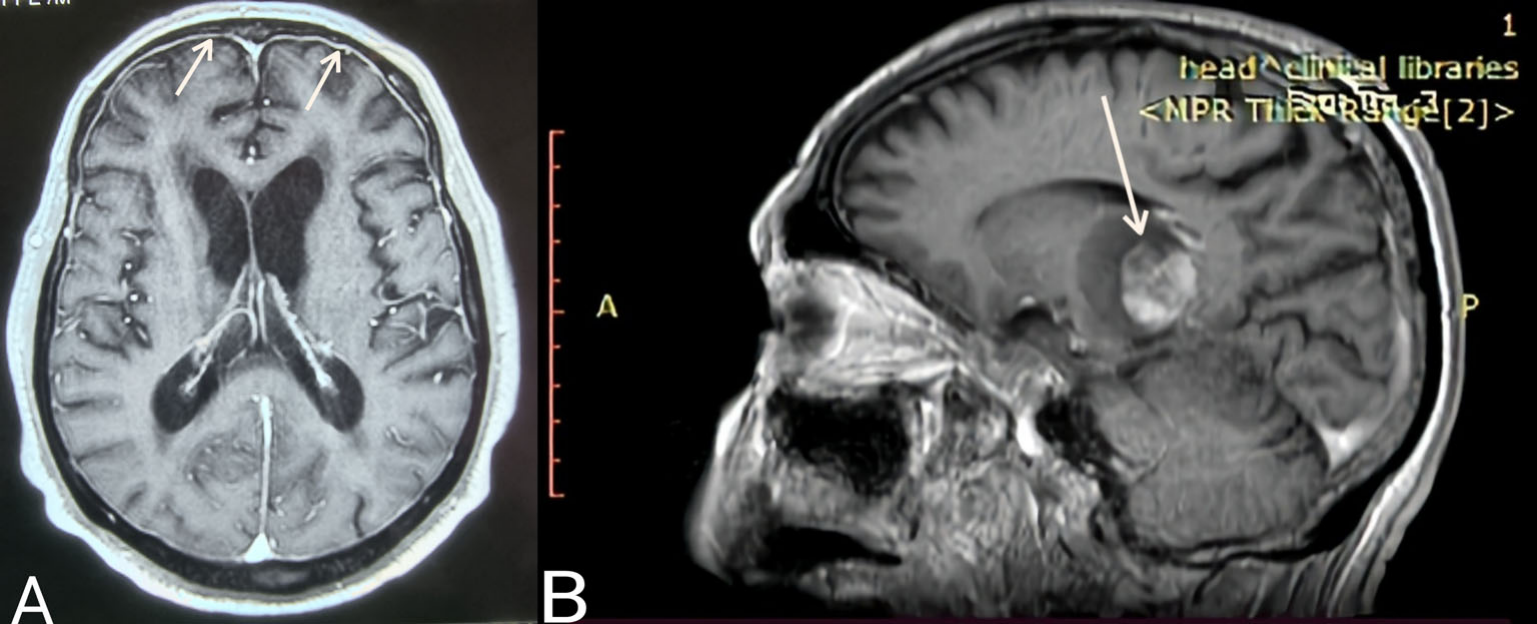
**(A)** Case 3: Axial T1 MR image (gadolinium enhanced) showing meningeal enhancement-diffuse form of BNS (white arrows). **(B)** Case 4: Sagittal T1 MR image (gadolinium enhanced) showing a brain mass- tumoral form of BNS (white arrow).

**Case 3.** The most uncommon presentation of BNS occurred in a 71-year-old man with a 12.5-year history of WM and multiple lines of therapy. Neurologists investigated the patient after he developed a bilateral hand tremor. He was diagnosed with Parkinson’s disease (PD) following their evaluation. His brain MRI at the time revealed periventricular white matter abnormalities. He had PD medication for nearly 2.5 years without improvement, until his worsening headaches prompted us to perform a lumbar puncture, which revealed a significant CSF pleocytosis of 59 cells/mm³, which flow cytometric analysis identified as LPCs. BNS relapsed after three years on ibrutinib, during which his tremor improved but never totally disappeared. His clinical picture was completed by the addition of diplopia and steadily worsening headaches. A subsequent brain MRI revealed modest meningeal enhancement (**Figure 1A**), whereas somnolence and stupor manifested at the disease’s last stage.

**Case 4** presented with severe gait abnormalities, progressive headaches, and dizziness, necessitating his hospitalization. As a result of the alarming clinical picture, neurosurgeons performed an MRI, which revealed a brain mass occupying the right optic thalamus and migrating to the right midbrain and pons (**Figure 1B**). A lumbar puncture was then conducted, which indicated CNS invasion by WM cells, establishing the BNS diagnosis less than a month after the neurological symptoms began.

The following patient (**Case 5**) was a 48-year-old male who presented to the emergency department complaining of acute unbalance and headaches that had been gradually worsening over the previous month. Diplopia was added to these symptoms. He was admitted to the neurosurgery department, where clinical examination revealed considerable muscle weakness in his lower extremities and hypesthesia at the L2 and L3 dermatomes. His gadolinium-enhanced brain MRI revealed leptomeningeal enhancement, while his lumbar spine MRI was normal. CSF investigation was performed in light of his WM history and revealed monotypic LPCs and the presence of the *MYD88 L265P* mutation in the CSF samples.

BNS was diagnosed most recently in a 61-year-old male (**Case 6**) who was diagnosed with asymptomatic WM in 2018. Since then, his disease had progressed gradually, with mild anemia, increasing IgM, and lymphadenopathy. Simultaneously, he began exhibiting bilateral upper limb weakness and cervical muscle spasms. Additionally, exophthalmos and edema of the eyelids were observed. On the basis of these findings, MRIs of the brain and viscerocranium were performed, revealing bilateral enlargement of the extraocular muscles due to LPCs infiltration.

The diagnosis of BNS in this short case series was made using CSF examinations (**Table 2**). Pleocytosis of the CSF was detected in all samples at a level of more than 30 cells/mm³ (range 33–400). The clonality of the B-lymphocytes in CSF fluid was confirmed by flow cytometry. Five patients exhibited an abnormally high quantity of CSF protein. At the molecular level, the *MYD88* mutation was found using quantitative PCR in the CSF specimen of case 5 whereas the IgH gene was not studied in any CSF sample.

Interestingly, imaging tests using MRI and Positron Emission Tomography/Computed Tomography (PET/CT) scans found that 50% of patients had extramedullary disease in sites other than the CNS, including the skin, bones, liver, lymph nodes, eyelids, and extraocular muscles. Further laboratory findings at the time of BNS diagnosis are presented in **Table 3**.

**Table 3:** Laboratory findings.

Case number	WBC (/μl) (ANC)	Ht (%)	Hb (g/dl)	PLT (/μl)	SPEP	IFE	IgM (g/l)	IgG (g/l)	IgA (g/l)	β_2_-M (mg/l)	Cryos
Case 1	6,510 (3,810)	41.8	14.3	173,000	pos	IgM-κ	6.72	8.85	1.57	2.48	negative
Case 2	6,030 (2,690)	33.7	10.6	309,000	pos	IgM-κ	38.70	3.65	0.26	2.14	NA
Case 3	5,230 (2,810)	35.4	11.8	245,000	pos	IgM-κ	56.70	1.53	0.28	4.28	NA
Case 4	10,030 (8,100)	41.0	15.1	145,000	pos	IgM-κ	16.90	4.18	0.12	1.93	NA
Case 5	6,030 (3,650)	42.9	14.9	310,000	pos	IgM-κ	3.40	4.52	0.26	1.88	negative
Case 6	7,800 (4,900)	31.6	9.9	265,000	pos	IgM-κ	33.20	35.80	0.52	11.50	NA

WBC, white blood cells; ANC, absolute neutrophil count; Ht, hematocrit; Hb, hemoglobin; PLT, platelets; SPEP, serum protein electrophoresis; IFE, serum immunofixation; IgM, immunoglobulin M; IgG, immunoglobulin G, IgA, immunoglobulin A; β_2_-M, beta2 microglobulin; Cryos, cryoglobulins; pos, positive (monoclonal band in gamma globulin region); NA, not available.

### Management

Only one patient was undergoing therapy for WM at the time of the BNS diagnosis. The majority of patients (5/6) were heavily pretreated (**Table 1**), with three median lines of therapy (range 1-5) and had either stable disease (n=2) or a progressively rising IgM level (n=3) at the time of BNS onset. At the time of therapy initiation for WM, one patient was diagnosed with BNS. At the time of BNS diagnosis, the median serum IgM level was 25.05 g/l (range 3.4-56.7 g/l).

Our standard therapeutic approach included a triplet intrathecal regimen (IT) consisting of methotrexate 12 mg, cytarabine 50 mg, and dexamethasone 4 mg (IT MTX, ARA-C, DEX) (**Table 2**). We administered four courses of the IT triplet and observed improvements in neurological symptoms and signs as well as clearance of the LPCs from the CSF. Three patients received subsequent intensive immunochemotherapy for a total of four cycles (range 2-4), which included rituximab, high-dose MTX, and ARA-C (R-Hi MTX/ARA-C) administered at 21-day intervals according to the Hyper-CVAD (hyperfractionated cyclophosphamide, vincristine, doxorubicin, dexamethasone) protocol (14). For patients with stable WM disease who could tolerate a cytotoxic regimen, this was the preferred treatment option. They all had a partial response (PR) that lasted an average of 33 months (range 10-58).

Rituximab in combination with bendamustine (R-Benda) was chosen as the initial regimen for two patients with BNS who required WM therapy concurrently. This regimen has shown activity in patients with BNS (9). Due to intolerance, one of them (case 5) received only one cycle of R-Benda and was then treated with ibrutinib, achieving a complete response (CR). The other patient (case 6) whose BNS was diagnosed at the time of treatment for symptoms of WM received 6 cycles of R-Benda, followed by ibrutinib. After 5 months on ibrutinib, his CNS disease resolved and he achieved a CR. Ibrutinib was administered to five patients (three as consolidation and two as an initial dose) at a dose of 420 mg daily until BNS or WM progression or intolerance developed. It was generally well tolerated, with the exception of a few manageable neutropenic episodes in two patients that necessitated its temporary discontinuation. There was no radiotherapy administered to any of the patients.

Two patients with BNS (cases 1 and 3) relapsed after 10 and 33 months of treatment, respectively. In all of these cases, the CNS disease progressed rapidly and there was no response to salvage IT or systemic therapy. After 40 months of ibrutinib treatment, one patient (case 2) developed a relapse of WM. Following that, he was then treated with R-Benda (6 cycles) and achieved complete remission. Twenty months later, his condition deteriorated, with PET/CT scans revealing abnormalities in his liver and bones. Biopsies from both sites revealed infiltration of WM LPCs, ruling out high grade lymphoma transformation. Subsequent lines of therapy, including R-Benda and Venetoclax were ineffective. **Table 2** contains information about BNS treatment and response.

Three patients have died as a result of WM and/or BNS progression. Overall survival for WM was 16.2 years (range 5.8-18.5) and for BNS was 3.5 years (range 1.2-7). After a median follow-up of 2.3 years (range 0.8-2.7) following BNS diagnosis, three patients are still alive and on ibrutinib (2 achieving CR, 1 achieving PR).

## Discussion

BNS is a rare manifestation of a rare disease affecting approximately 1% of patients with WM. Six patients (7%) were identified with BNS in our data set following a thorough evaluation of all WM patients’ records, revealing a substantially higher rate of BNS cases than previously reported (3, 4). Despite our modest sample size, many of our findings corroborate the results from the two largest multi-institutional studies (6, 7). Similarities were detected in terms of age at BNS diagnosis, male sex prevalence, the likelihood of a late BNS onset, and BNS treatment heterogeneity.

Although our department is a referral center for plasma cell neoplasms, these BNS cases most likely reflect a “real world” incidence of an unquestionably under-diagnosed condition. Interestingly, all BNS diagnoses have been made in the recent decade, whereas none had been made before. This finding could be better interpreted as a misdiagnosis of BNS in the past rather than a shift in the epidemiology and biology of the disease.

BNS transcends the simple definition of CNS involvement in WM. It can develop at any time point over the course of WM (15), regardless of whether the disease is active or amenable to therapy. As noted above, its symptoms may mirror those of other diseases and disorders (e.g., Parkinson’s disease, transient ischemic attack, seizures) resulting in significant delays in diagnosis and treatment. This was highly significant in three of our patients, who were diagnosed 19, 24 and 30 months after the initiation of symptoms. Before receiving a diagnosis, these patients were seen by at least 3-5 physicians, including general practitioners, internists, neurologists, orthopedic surgeons, and cardiologists. Additionally, they underwent a plethora of unnecessary treatments (e.g. aspirin, antiepileptics, antiparkinson agents). Notably, they were all diagnosed prior to 2016, when vigilance and attention to BNS were relatively low. On the contrary, among individuals diagnosed after 2019, the time between BNS onset and diagnosis was less than 6 months. This most probably reflects an increase of awareness of the syndrome among physicians over time, indicating that our understanding of WM has progressed. In view of the above, we strongly support that BNS was and continues to be underdiagnosed.

There is a high degree of clinical overlap between BNS and other WM complications, and even experienced clinicians struggle to differentiate them. As an example, we discussed a patient (case 2) who presented with blurred vision and a headache, both of which could have been caused by hyperviscosity, a condition associated with WM (16). Notably, in one patient (case 1), the median and ulnar nerve impairments were the most prominent symptoms at BNS onset, indicating the presence of mononeuropathy or peripheral neuropathies that can be seen in WM. However, as the patient’s clinical picture deteriorated, resulting in foot drop, the initial diagnosis changed and the belief that it was a peripheral BNS became stronger, as previously stated. BNS with coexisting peripheral nerve damage has been previously documented in the literature by Lunn et al., suggesting that LPCs can also penetrate the blood-nerve-barrier, and this may be the case for our patient (17).

The differential diagnosis of BNS is very broad due to similarities with disorders both within and outside the spectrum of WM. Thus, tools for precise and early diagnosis are important. In a recent review, Castillo et al. described the presence of WM cells in CSF as the “gold standard” for diagnosis (4). All diagnoses in our series were made on the basis of CSF analysis, which was performed on all patients with suspicious clinical and radiological findings. Because the majority of patients had nonspecific MRI signals, imaging was only partially helpful in diagnosing BNS. According to Simon et al., MRI can be used not only for diagnosis but also for decision-making regarding treatment initiation and evaluation of therapeutic response. Additionally, he recommended repeating the CSF assay and MRI in cases of high clinical suspicion but negative results (6).

Following the seminal paper by Treon et al. (18) on the discovery of the recurrent mutated *MYD88 (L265P)* gene, there is now a better understanding of the prosurvival signaling this mutation mediates in WM cells *via* BTK and other molecules. It also facilitated the development of BTKi and enabled for a paradigm shift toward targeted treatment in WM. Ibrutinib was the first oral BTKi to be approved for WM, with an extremely high efficacy rate (19, 20). It is also a major game-changer for patients with BNS because it has been proved to be effective in CNS involvement (13). Thus, several case reports, retrospective studies, and reviews rekindled the interest in BNS. Ibrutinib has a high level of brain distribution, as evidenced by papers studying its pharmacokinetics, which report rapid blood-brain barrier (BBB) penetration at a median period of 0.29 hours (range 0.2-0.32 hours) (21). Zanubrutinib, the second BTKi licensed for the treatment of WM, has demonstrated a favorable outcome in patients with MYD88 wild-type WM (22). Additionally, it revealed considerable improvement in neurological impairments and MRI abnormalities in a patient with BNS, thereby expanding therapeutic choices for those experiencing this rare complication of WM (23).

Precision diagnosis of BNS in the ibrutinib era and beyond will revolutionize how we treat this complication by improving treatment quality and minimizing unnecessary hospitalizations and toxicity. BTKi have the potential to dramatically transform the care of BNS by replacing the intensive chemotherapy regimens now utilized in primary CNS lymphoma. Castillo et al. have published a retrospective study demonstrating the efficacy of ibrutinib in the management of BNS (13). Ibrutinib was given upfront in 39% of the patients, while the remainder had received at least one prior therapy (range 1-5). The response rate (CR and PR) was 41%, although non-responders had clinical improvement. The estimated 2-year survival rate after initiating ibrutinib was 81% and the estimated 5-year survival rate after BNS diagnosis was 86%, both of which demonstrate promise in the management of BNS. In our small case series of five patients treated with ibrutinib, 40% attained CR and 60% achieved PR.

As stated in this article, the goals for patients with BNS should be a sustained reduction in symptom burden and prevention of progression, not a rapid CR (9). We believe that ibrutinib monotherapy is the optimal treatment option for individuals with a leptomeningeal type of BNS, as it can induce sustained remissions. Additionally, it is the best option for people who are susceptible to serious and unpredictable adverse events associated with high-dose chemotherapy. A brief course of intense immunochemotherapy, with a preference for methotrexate-based regimens, followed by ibrutinib, may be recommended for patients with brain masses. Our patient (case 4) is a real-world example of someone who was effectively treated with the aforementioned therapy and acquired a long-lasting PR lasting more than 26 months. However, ibrutinib has shown a significant success in the management of tumoral forms of BNS when used alone, indicating an exceptional activity against this uncommon manifestation of WM (24). Bendamustine is also an option if systemic disease control is essential and the central nervous system is involved (9).

Additional large-scale research is required to elucidate on numerous aspects of BNS, including risk and predictive variables, early and precise diagnosis and management, and perhaps prevention. The latter appears plausible, as we anticipate a significant increase in the number of patients with WM treated upfront with ibrutinib or another BTKi in the coming decades. Unlike conventional chemotherapy regimens, which are only effective for a limited number of cycles, ibrutinib allows for long-term per os administration with a good safety profile and excellent BBB penetration. As a result, it may completely alter the occurrence of BNS by impairing LPCs homing in the CNS in the first place. However, the precise pathogenesis, pathophysiology, and risk factors for BNS remain unknown, and any idea addressing its prevention requires additional research.

This retrospective study helped us in the clarification of different elements of BNS in WM disease. As previously stated, BNS presents numerous diagnostic challenges: early recognition of neurological symptoms, the requirement for an accurate and timely diagnosis, the diagnosis of asymptomatic patients or patients with atypical presentations, and the limitations of the available imaging studies and molecular tests (i.e. false negative brain MRI in CNS involvement). When it does manifest, BNS is a debilitating neurological disorder associated with a high rate of impairment and comorbidity. Prompt treatment initiation is crucial for the best potential outcome, but requires an accurate and timely diagnosis.

## Data Availability Statement

The raw data supporting the conclusions of this article will be made available by the authors, without undue reservation.

## Ethics Statement

The current study was reviewed and approved by the Scientific and Ethical Committee of AHEPA University Hospital. Written informed consent was obtained from the individual(s) for the publication of any potentially identifiable images or data included in this article.

## Author Contributions

DK collected, analyzed the data and drafted the initial manuscript. SC, AA, AX, AR, and CF collected and analyzed data. EH designed the study, provided and analyzed the data, supervised research, and wrote the manuscript. All authors contributed to the article and approved the submitted version.

## Conflict of Interest

EH has received honoraria from Janssen.

The remaining authors declare that the research was conducted in the absence of any commercial or financial relationships that could be construed as a potential conflict of interest.

## Publisher’s Note

All claims expressed in this article are solely those of the authors and do not necessarily represent those of their affiliated organizations, or those of the publisher, the editors and the reviewers. Any product that may be evaluated in this article, or claim that may be made by its manufacturer, is not guaranteed or endorsed by the publisher.
