# Supplementary material for: Bing-Neel Syndrome: Real-Life Experience in Personalized Diagnostic Approach and Treatment
Source: Front Oncol. 2022 Jun 29;12:891052. doi: 10.3389/fonc.2022.891052 (PMC9278058; doi:10.3389/fonc.2022.891052)
Supplement: Supplementary file 1 [file Table_1.pdf]

Supplementary Table 1. "Systemic disease history"

Cases	Age at WM diagnosis (Year of WM diagnosis)	Reason for treatment initiation	Prior therapy for WM and response
Case 1	62 (2009)	Anemia (Hb: 9.3 g/dl)<br>Thrombocytopenia (PTL: 45,000/µl)<br>Splenomegaly<br>Hyperviscosity symptoms (headache, dizziness)	1st line (2009): R-CHOP ×4 (VGPR)<br>2nd line (2012): Rituximab/weekly ×4 (SD)
Case 2	40 (2004)	Anemia (Hb: 9 g/dl)	1st line (2004): DRC ×8 (PR)<br>2nd line (2007): Rituximab/weekly ×4 (SD)<br>3rd line (2010): CPA+Dex ×7 (PR)<br>4th line (2012): DRC ×6 (PR)<br>5th line (2013): Rituximab/monthly ×4 (PD)
Case 3	56 (2001)	Hyperviscosity symptoms (mucosal bleeding)	1st line (2001): Rituximab/weekly ×8 (SD) (with a 2-month discontinuation in between)<br>2nd line (2002): Chlorambucil/28days ×10 (PR)<br>3rd line (2006): DRC ×4 (PR)<br>4th line (2009): DRC ×6 (PR)<br>5th line (2011): FC ×5 (PR)<br>6th (2013): R-CEOP ×4 (PR) (discontinuation due to neutropenia)
Case 4	61 (2017)	Increasing IgM (76.7 g/l)<br>Splenomegaly	1st line (2017): FC (and R added after IgM reduction) ×6 (PR)
Case 5	33 (2004)	Anemia (Hb: 7.8 g/dl)<br>Increasing IgM (117 g/l)<br>Hyperviscosity symptoms (dizziness, tinnitus, mucosal bleeding)	1st line (2004): Plasmapheresis ×6, DRC ×8 (PR)<br>2nd line (2011): CEOP ×3 (MR)<br>3rd line (2011): Plasmapheresis ×3 plus FCR ×6 (VGPR)
Case 6	58 (2018)	Anemia (Hb: 9.7 g/dl)<br>BNS	No prior treatment (patient was diagnosed with BNS at the time of treatment initiation for WM)

WM: Waldenström's Macroglobulinemia; BNS: Bing-Neel syndrome; Hb: hemoglobin; PTL: platelets; R-CHOP: rituximab- cyclophosphamide, doxorubicin hydrochloride, vincristine, prednisone; DRC: dexamethasone, rituximab, cyclophosphamide; CPA: cyclophosphamide; Dex: dexamethasone; FC: fludarabine, cyclophosphamide; R-CEOP: rituximab, cyclophosphamide, etoposide, vincristine, prednisone; IgM: immunoglobulin M; VGPR: very good partial response; PR: partial response; MR: minor response; SD: stable disease; PD: progressive disease
